# Commentary: Visual attention is not deployed at the endpoint of averaging saccades

**DOI:** 10.3389/fpsyg.2018.02166

**Published:** 2018-11-09

**Authors:** Stefan Van der Stigchel, Jelmer de Vries

**Affiliations:** Experimental Psychology, Helmholtz Institute, Utrecht University, Netherlands

**Keywords:** eye movements, saccades, attention, vision, action

When awake, we constantly interact with our visual surroundings. To guide us, our behavior is selective: Rather than processing all objects to the same extent, we prioritize objects of interest. Primarily, this is achieved by *overt* attentional shifts: We sequentially focus the fovea on objects of interest by moving our eyes. However, locations can also be prioritized by shifting attention *covertly*, without physical movement. The premotor theory of attention hypothesizes that both these attentional shifts are driven by our oculomotor system (Rizzolatti et al., 1987, 1994). Specifically, a covert attentional shift is suggested to be a direct result of the preparation of an overt eye movement. The hallmark theory finds bases in early physiological studies which observed that visually-responsive neurons in the superior colliculus react more vigorously toward spots of light when they are the destination of an upcoming saccade (Wurtz and Mohler, 1976) and demonstrations that saccades are consistently preceded by a covert attentional shift (Kowler et al., 1995; Deubel and Schneider, 1996).

In a recent publication in *PLoS Biology*, Wollenberg et al. (2018) show that the attentional locus *can* be dissociated from the saccade endpoint, arguing that this finding conflicts with the premotor theory of attention. Interestingly, earlier we observed the same finding in our paper in *Journal of Vision* (Van der Stigchel and de Vries, 2015), but found it consistent with the premotor theory. Both studies employ a highly similar global effect paradigm, where placing targets in close proximity results in saccade endpoints deviating from the intended targets toward the intermediate location. Both studies find that for accurate, as well as averaging saccades, attention is focused on the target locations and not on the intermediate location. Thus, in case of averaged saccades the saccade endpoint is not the locus of attention.

In distinguishing their observations from our findings, Wollenberg and colleagues refute our study incorrectly. Our findings are discarded stating they “*most likely reflect a masking effect”* of the probe at the intermediate location, disregarding our work to control for this confound. To ensure masking strength was similar at all locations, extensive piloting led to a design where probes used to determine the attentional locus were each embedded in a placeholder ring. Also, the contrast of the rings alternated between positive and negative to minimize the influence of salience. Importantly, in our manuscript, threshold measurements showed that the intermediate location was actually associated with the lowest threshold. This means that, if anything, sensitivity for the probe at the intermediate location was facilitated rather than impaired.

Moreover, Wollenberg and colleagues state that we found no influence of the saccade endpoint on the allocation of attention, showing no presaccadic shift of attention. While indeed we do not report a direct comparison on this matter, we did note that “*only in the target probe condition did we see facilitation toward the target*.” However, in contrast to the direct comparisons executed by Wollenberg and colleagues, our repeated measures combined a number of probe conditions. Isolating performance at the location of the target (divided over three bins to more closely resemble Wollenberg's design) a *post-hoc* test does show improved performance for saccades landing closer to the target (repeated-measure ANOVA: *p* = 0.017).

These corrections aside, most importantly, we believe Wollenberg and colleagues have misinterpreted the premotor theory. The finding that visual attention is not directly coupled to the saccade endpoint does not refute the premotor theory. The theory does not predict a direct coupling between attentional selection and saccade *endpoint*, but a coupling between attention and oculomotor *programs (“Attention is simply deployed to a given point in accordance to the parameters of the motor program,”* Rizzolatti et al., 1987). Many studies observed that visual attention and saccadic endpoints can be dissociated (Deubel and Schneider, 1996; Doré-Mazars et al., 2004). However, because the saccade endpoint does not necessarily reflect the outcome of a single oculomotor program, a dissociation between endpoint and attentional locus does not invalidate the premotor theory.

To refute the pre-motor theory, it would have to be demonstrated that attention is dissociated from the saccade endpoint, while the latter aligns with an oculomotor program. It is difficult to argue that this requirement is satisfied using the global effect. For good reason the phenomenon is often referred to as *saccade averaging*: It is thought to be a motor phenomenon where competing oculomotor programs result in an *averaged saccade* (Van der Stigchel and Nijboer, 2011; Bhutani et al., 2012). Various models of saccade generation include competitive interactions between neurons coding for possible target locations on a common motor map (Trappenberg et al., 2001; Meeter et al., 2010). Here saccade targets are represented by individual peaks of activity. The endpoint is determined by the weighted average of this activity at the moment the eye movement is initiated.

The motor map in which this competition is resolved is often thought to be located in the retinotopically-organized motor layers of the superior colliculus (SC) (Sparks and Hartwich-Young, 1989; Schall, 1991). Neurophysiological recordings in the deep layers of the SC have shown that the highest neural activity is positioned at the sites of the two visual stimuli in trials in which the global effect was observed (Edelman and Keller, 1998). Wollenberg and colleagues suggest that their findings may resemble saccades in a report that did find greater activity between the targets (Glimcher and Sparks, 1993). However, as has been argued previously, the mere 5% averaging saccades in this study may not reflect typical averaging saccades (Edelman and Keller, 1998). The observation that attentional allocation was strongest at the possible saccade targets is therefore in line with the evidence that the highest neural activity in the SC remains at the sites of the two visual targets during averaging saccades and does not conflict with the pre-motor theory which states: “*Spatial selective attention is a consequence of an activation of neurons located in the spatial pragmatic maps”* (Rizzolatti et al., 1994).

Taken together, we argue that Wollenberg's findings are insufficient to invalidate the premotor theory of attention. Whereas, the saccadic endpoint is determined by the average of the active oculomotor programs, the attentional shifts may follow the locations of the active oculomotor programs. For a revision of the premotor theory a simple dissociation of saccade endpoint and attentional locus is insufficient; one also has to demonstrate that the endpoint and the oculomotor program coincide.

## Author contributions

All authors listed have made a substantial, direct and intellectual contribution to the work, and approved it for publication.

### Conflict of interest statement

The authors declare that the research was conducted in the absence of any commercial or financial relationships that could be construed as a potential conflict of interest.
